# Recent developments in biomarkers in Parkinson disease

**DOI:** 10.1097/WCO.0b013e3283633741

**Published:** 2013-07-03

**Authors:** Anthony H.V. Schapira

**Affiliations:** Department of Clinical Neurosciences, UCL Institute of Neurology, London, UK

**Keywords:** alpha-synuclein, biomarker, genes, imaging, Parkinson disease

## Abstract

**Purpose of review:**

Parkinson disease is the second most common neurodegenerative disease after Alzheimer disease, and current demographic trends indicate a life-time risk approaching 4% and predict a doubling of prevalence by 2030. Strategies are being developed to apply recent advances in our understanding of the cause of Parkinson disease to the development of biomarkers that will enable the identification of at-risk individuals, enable early diagnosis and reflect the progression of disease. The latter will be particularly important for the testing of disease-modifying therapies. This review summarizes recent advances in Parkinson disease biomarker development.

**Recent findings:**

Recent reports continue to reflect the application of a variety of clinical, imaging or biochemical measurements, alone or in combination, to general Parkinson disease populations. Probably the most promising is the assay of alpha-synuclein in the diagnosis and evolution of Parkinson disease. At present, detection techniques are still being refined, but once accurate and reproducible assays are available, it will be important to define the relationship of these to early diagnosis and progression. Alpha-synuclein concentrations may also be modulated by certain disease-modifying agents in development and so may represent a measure of their efficacy. It has to be accepted that no single measure currently fulfils all the necessary criteria for a biomarker in Parkinson disease, but combinations of measures are more likely to deliver benefit.

**Summary:**

The Parkinson disease biomarker field is approaching a stage when certain combinations of clinical, imaging and biochemical measures may identify a proportion of individuals at risk for developing the disease. However, their general applicability may be limited. Attention is now turning to stratification of Parkinson disease into certain at-risk groups defined by genotype. The application of multimodal screening to these populations may be more rewarding in the short term.

## INTRODUCTION

The Biomarkers Definitions Working Group has defined a biomarker (or a biological mark) as a characteristic that can be objectively measured and evaluated as an indicator of normal biological processes, pathogenetic processes or pharmacologic responses to a therapeutic intervention.

In the broadest sense, a biomarker may thus be a quantifiable clinical evaluation, a biochemical assay or an image parameter. A biomarker for Parkinson disease may be useful to:

1. Aid in diagnosis and the distinction of parkinson disease from other Parkinsonian diseases such as multiple system atrophy, progressive supranuclear palsy and so on.
2. Aid in the early diagnosis of Parkinson disease, preferably in the prodromal premotor period, that is before a clinical diagnosis can be made.
3. Provide a means by which to follow disease progression.
4. Use in disease modification trials as a means to assess efficacy of an intervention. For this, the biomarker would need to reflect disease progression in a pathway targeted by the study drug, or be a more general indicator of disease progression.

**Box 1:**
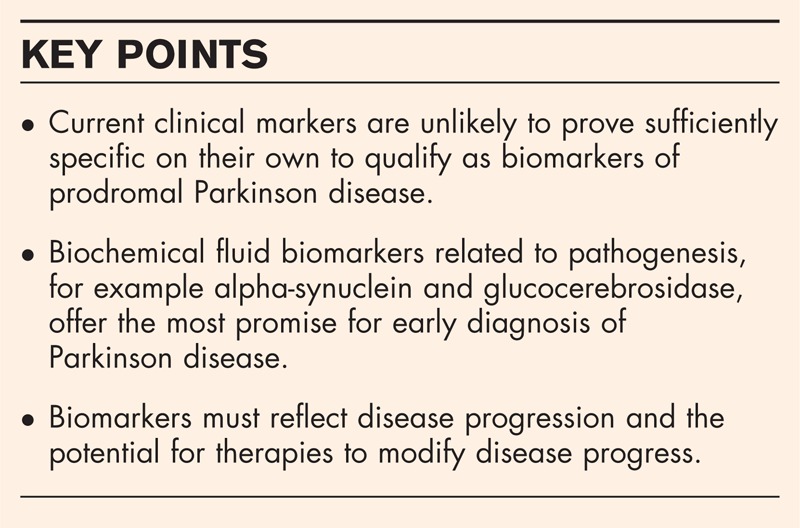
no caption available

Currently, it seems unlikely that any single evaluation will be capable of fulfilling any or all of these roles, but the hope is that a combination of biomarkers might achieve some.

The field of biomarkers in Parkinson disease has attracted significant attention and there have been numerous contributions to the field in the recent past, and even since the most recent review of this topic for *Current Opinion in Neurology*[1]. This review will focus on only the most recent developments. Most references are confined to 2011 and subsequently, and the reader is referred to a number of excellent reviews of this topic for additional references [2^▪^,^3^,4^▪^,^5^,^6^].

## CLINICAL MARKERS

The motor features of Parkinson disease have defined the disease and remain the most important diagnostic marker [7]. However, there are some important limitations to their application as a biomarker according to the requirements summarized above. Bradykinesia, rigidity and tremor are thought to appear only when the loss of dopaminergic neurones is advanced, so they cannot be used as an early marker. The motor features progress, but at variable rates in different individuals [8]. Their quantification is reasonably reproducible [9], but may be limited by floor effects, especially in early disease [10]. Although a reasonably accurate reflection of disability and response to symptomatic treatment, motor features cannot be used as a marker of disease modification if the interventional agent has a symptomatic effect, unless a modified trial design is used such as a delayed start.

Nonmotor clinical features are common in Parkinson disease and may often arise even before motor symptoms [11]. Thus, hyposmia, rapid eye movement sleep behaviour disorder (RBD), depression, autonomic dysfunction and constipation have all been linked to the clinical and pathological evolution of Parkinson disease. The sensitivity, specificity, positive and negative predicted values of these clinical features have recently been reviewed [3]. However, these nonmotor features are a mixture of risk factors and biomarkers, the former highlighting a symptom, sign or measure that indicates the index individual is at a greater risk of developing Parkinson disease than a control group without the corresponding feature(s), whereas the latter is an objective measure, usually directly related to pathogenesis.

## PATHOLOGICAL MARKERS

Recent data have drawn attention to the presence of alpha-synuclein (SNCA) and Lewy bodies in the gut of patients with Parkinson disease in biopsies obtained several years before the onset of motor features [12^▪^]. This very important observation requires confirmation with larger studies, but could represent an interesting and relatively minimally invasive technique for premotor assessment and potentially as a marker for the efficacy of agents designed to reduce SNCA levels that have access to systemic tissues.

## IMAGING MARKERS

18-Fluoro-dopa positron emission tomography (PET) or various dopamine presynaptic markers, for example dopamine transporter (DAT) or vesicular monoamine transporter type 2, identified by single photon emission tomography (SPECT), fulfil many of the requirements of a useful marker of Parkinson disease. They reflect the loss of nigrostriatal dopamine neurons that cause the typical early motor features of Parkinson disease. The scans may be abnormal before the motor signs appear and they progress as neurodegeneration continues. However, the correlation with the Unified Parkinson Disease Rating Scale is limited, especially in the early stages of the disease [13]. One study using SPECT recently reported correlation of severity of DAT deficit at baseline with progression of clinical features over 22 months [14]. These imaging modalities do not reliably distinguish between different neurodegenerative parkinsonian syndromes, and they may be modulated by dopaminergic drug interventions: levodopa causes a decline in both PET and SPECT. Thus, they are considered of limited use as markers, although are useful as diagnostic tools in certain circumstances.

I-123-metaiodobenzylguanidine (MIBG) with cardiac SPECT identifies postganglionic sympathetic denervation and is decreased in patients with Parkinson disease and other synuclein diseases. It can be an early indicator of catecholaminergic dysfunction.

Transcranial sonography (TCS) can reveal increased echogenicity in up to 90% of patients with clinical Parkinson disease. A recent prospective study of asymptomatic individuals over 37 months showed that 0.7% developed Parkinson disease and the presence of nigral hyperechogenicity at baseline increased the risk for Parkinson disease by 17-fold [15]. However, TCS has limitations in terms of specificity and positive predictive value. Also, it is not known what the hyperechogenicity reflects and it does not seem to evolve or progress with disease. Nevertheless, further work on TCS will be helpful, as it is a noninvasive, cheap and accessible technique that could find its value in combination with other Parkinson disease markers.

Advanced techniques of MRI including diffusion weighted imaging may be useful to identify a proportion of patients with progressive supranuclear palsy or multiple system atrophy from Parkinson disease. High field strength magnetic resonance and fractional anisotropy with diffusion tensor imaging are under evaluation for the diagnosis of Parkinson disease [16,17]. Reduced fractional anisotropy in substantia nigra correlated with motor severity and offers a particularly promising tool that may correlate with disease progression. MRI techniques to measure brain iron levels have shown promise in their application to Parkinson disease. A recent study [18] measured nigral (compacta and reticulata) and caudate iron over 3 years and showed accumulation in all these structures in patients with Parkinson disease over time, which correlated with motor progression.

## BIOCHEMICAL MARKERS

The development of biochemical markers for the early diagnosis and progression of Parkinson disease is most logically based on an understanding of disease pathogenesis. Several pathogenetic pathways are considered relevant including mitochondrial dysfunction, oxidative stress, inflammation and protein accumulation, aggregation and propagation [19,20]. The identification of prospective markers has also been informed by advances in the genetics of Parkinson disease, for example the use of SNCA or DJ-1.

### Alpha-synuclein

SNCA expression and aggregation as Lewy bodies are considered central to the pathogenesis of Parkinson disease. Thus, the detection of this protein may enable correlation with the risk and progression of the disease. SNCA may be transmitted between neurons and so has access to the extracellular space, although this may be in exosomes. SNCA has been detected in plasma, saliva and cerebrospinal fluid (CSF). There are significant issues relating to the reproducibility of detection techniques from different sources, but most have demonstrated a reduction in SNCA in CSF in Parkinson disease and other parkinsonian syndromes [21,22]. The expression of data as a ratio of oligomeric to total SNCA may be most valuable. In one analysis this ratio reached 89.3% sensitivity and 90.6% specificity for Parkinson disease diagnosis [23]. Two additional studies support the application of the oligomer:total SNCA ratio [24,25]. Alternatively, 129-phosphorylation posttranslational modification of SNCA, the most abundant form in Lewy bodies, may be a useful target for detection [26]. The ratio of total SNCA with 129-phosphorylated SNCA in CSF improved discrimination of Parkinson disease from other parkinsonisms [27].

Why SNCA levels should be lower in Parkinson disease CSF is not clear, but may reflect alterations in transcription, splicing or processing, or increased clearance. The relationship of SNCA levels to the stage of disease and its evolution over time, or the effect of symptomatic therapies is not yet known. The effects of therapies to influence SNCA expression and turnover, including immunotherapies designed to reduce CNS SNCA, could in part be informed by CSF SNCA levels.

SNCA and tau interact to promote mutual aggregation. Thus, it has been suggested that simultaneous measurement of both proteins in CSF may be of value in Parkinson disease. This is supported by recent data enabling the distinction of synucleinopathies from other neurodegenerative diseases [21,28].

Several studies have sought to measure SNCA in blood cells, plasma and saliva, but the results have been variable and, as with CSF studies, this probably reflects the different assay techniques used.

### DJ-1

Mutations of DJ-1 are a rare cause of parkinsonism. The full repertoire of functions of DJ-1 is unknown but probably includes oxidant signalling with mitochondria. CSF and plasma levels of DJ-1 have been found to be unaffected, elevated or more recently reduced compared with controls [29,30]. There was no correlation between CSF DJ-1 and fluoro-dopa PET in eight patients with *LRRK2* mutations compared with controls [31].

### Other markers under investigation

There has been a wide range of potential protein markers investigated in Parkinson disease. These include neurofilaments, interleukins, osteopontin and hypocretin. However, results have been negative or inconclusive. A very recent study has identified low levels of plasma ApoA1 as a significant risk factor for Parkinson disease, and concentrations correlated with increased putaminal loss on DAT scan [32^▪▪^]. ApoA1 is a major component of high-density lipoprotein (HDL), and it is of note that statin use, which increases HDL, is associated with a reduced risk for Parkinson disease [33].

CSF amyloid-beta(1–42) levels have been studied to determine whether they reflect the risk for dementia in Parkinson disease. In Alzheimer disease, there is an increase in CSF total tau and phosphorylated tau, with a reduction in amyloid-beta(1–42). In Parkinson disease, however, tau levels appear unchanged with only a small decrease in amyloid levels [34]. However, there is interest in using amyloid levels and tau ratios to predict cognitive decline in Parkinson disease [35–39]. Low levels of plasma epidermal growth factor have been reported to correlate with cognitive function in Parkinson disease and to be a marker for future cognitive decline [40].

Urate is an antioxidant and studies have shown that the risk of Parkinson disease is inversely proportional to plasma urate [41,42], and levels are lower in patients with Parkinson disease [43]. However, its use as a diagnostic marker in isolation is limited.

## STRATIFICATION OF PARKINSON DISEASE

Parkinson disease has multiple causes and so the biomarker studies performed to date have almost all been on a heterogeneous population in aetiological terms. This inevitably limits the performance of biomarkers related to specific pathogenetic pathways. However, the identification of certain genetic causes of Parkinson disease has enabled the characterizsation of specific more homogeneous subgroups. These include those with *LRRK2* mutations, who represent approximately 0.5–1.0% of unselected Parkinson disease cases in the general western communities, although considerably greater proportions of familial Parkinson disease or those of Ashkenazi Jewish origin. Studies are currently underway to determine whether there are specific biochemical or imaging profiles that reflect disease expression and progression in both *LRRK2* Parkinson disease patients and asymptomatic carriers. Of course, markers that may reflect these processes in *LRRK2* Parkinson disease may also be applicable to non-*LRRK2* Parkinson disease.

Patients with mutations of the glucocerebrosidase (*GBA*) gene represent the largest identifiable group of individuals at risk for Parkinson disease. The precise risk for Gaucher disease patients developing Parkinson disease is not known, but has been variously estimated as 20–30 fold [44,45]. Conversely, 5–10% of patients with Parkinson disease have *GBA* mutations, making these mutations numerically the most important risk factor for the disease identified to date. Parkinson disease associated with *GBA* mutations (GBA-PD) is clinically, pathologically and pharmacologically indistinguishable from idiopathic ‘sporadic’ Parkinson disease, although GBA-PD has a slightly earlier onset (∼5 years) and rather more frequent cognitive dysfunction. Recent studies demonstrating a reciprocal relationship between SNCA and the *GBA* enzyme (glucocerebrosidase) are of considerable importance to our understanding of the pathogenesis of GBA-PD and idiopathic Parkinson disease [46,47]. The pathogenesis of the neurodegeneration in this group in this group appears to mirror pathways identified in non-*GBA* positive Parkinson disease, including mitochondrial dysfunction and oxidative stress [48] and lysosomal dysfunction [49]. Thus, they represent a large cohort not only for which specific therapies may be applicable [50] but also for detailed analysis for biomarkers within the lysosomal pathways that may be applicable to the general Parkinson disease population.

## OMICS

The investigation of biological specimens for biochemical profiles is entering a new era with the application of novel technologies capable of mass analyses. These include transcriptomics, proteomics and metabolomics. These powerful tools have the capacity to identify small changes in mRNA, protein or metabolite profiles between different cohorts and can be applied to tissue, cells or fluids [51]. Although powerful techniques, there remain issues with reproducibility of data. In part, this is likely to reflect the problem with population heterogeneity described above. A single biochemical profile is unlikely to reflect a complex disease process that may well evolve over time and be different between aetiological groups. Perhaps the most effective use of these technologies in the first instance will be their applicability to stratified groups of patients with Parkinson disease, with subsequent evaluation of any promising profiles in the general Parkinson disease population.

## BIOMARKER COMBINATIONS

There is a general consensus that it is unlikely that one single measure will faithfully reflect the complex pathological processes that underlie the development, expression and progression of Parkinson disease. It is much more likely that a combination of markers will be required and this may include clinical, for example olfactory function, biochemical and imaging markers to define at-risk individuals, early Parkinson disease diagnosis and progression. There have, of course, already been a number of attempts to do this particularly using clinical parameters, for example olfactory function, sleep behaviour and imaging in both general Parkinson disease populations [52,53] and genetically defined populations [54]. Multiple CSF markers have been analysed to differentiate Parkinson disease from other parkinsonian syndromes, dementia with Lewy bodies, Parkinson disease with dementia and Alzheimer disease, with some success [30,55].

The PARS (Parkinson At-Risk Syndrome study) has recruited 4999 patients with Parkinson disease who returned a screening olfaction test and questionnaire, and 669 were at or below the 15th centile for olfaction [56^▪^]. Hyposmia aggregated with other nonmotor features. This study involves an imaging arm and represents an important development in risk assessment and early clinical features for Parkinson disease.

## PRACTICAL USE OF BIOMARKERS IN PARKINSON DISEASE: A PERSONAL VIEW

It is often recited that even an early clinical diagnosis of Parkinson disease is made at a time of advanced nigral neurodegeneration. This is true, and indeed the rate of early loss of motor function that follows is rapid. It stands to reason that any disease-modifying agent with neuroprotective or neurorescue properties [57] will have most chance of success if given at the earliest period in the evolution of the neurodegenerative process [58]. However, an opportunity to slow or halt progression even at the time of Parkinson disease diagnosis would leave patients substantially improved over time. Thus, although earlier (premotor) diagnosis is a laudable goal, the more pragmatic view is to have a marker of disease progression against which putative neuroprotective or disease-modifying agents may be tested. This would represent a major advance in Parkinson disease research. Of course, these two ambitions are not mutually exclusive.

## CONCLUSION

The search for an appropriate biomarker for Parkinson disease continues. It is increasingly unlikely that any single measure will suffice, at least in the foreseeable future, and that a combination of measures will be required. Pragmatically, the most critical attribute for a biomarker is not that it diagnoses Parkinson disease early, before motor features appear, but rather that it reflects a pathogenetic process and progression of the disease against which potential disease-modifying agents may be judged.

## Acknowledgements

This work was supported in part by the Wellcome Trust/MRC Joint Call in Neurodegeneration award (WT089698) to the UK Parkinson's Disease Consortium (UKPDC) and Kattan Trust. A.H.V.S. is a NIHR senior investigator.

### Conflicts of interest

The author has received honoraria for consultancy and educational symposia from BI, Teva-Lundbeck, Orion-Novartis, UCB, Merck, Zambon and GSK.

## REFERENCES AND RECOMMENDED READING

Papers of particular interest, published within the annual period of review, have been highlighted as:

▪ of special interest
▪▪ of outstanding interest

Additional references related to this topic can also be found in the Current World Literature section in this issue (p. 453).
